# Military anti-shock garment: Historical relic or a device with unrealized potential?

**DOI:** 10.4103/0974-2700.43181

**Published:** 2008

**Authors:** Fatimah Lateef, Tan Kelvin

**Affiliations:** Department of Emergency Medicine, Singapore General Hospital, Outram Road, 1 Hospital Drive, Singapore – 169608

**Keywords:** Auto transfusion, hypotension, military anti-shock trousers, shock, tamponade effect

## Abstract

Military anti-shock trousers represents a medical device which has engendered very divergent views, even up to today. From the time the concept was formulated in 1903 by surgeon George W Crile, there have been significant swings in opinion and evidence. The guidelines, where available, are often kept relatively general and cautious. As a spin-off to the mechanism and technology, several alternative devices have been proposed or developed over the years. This include the auto-transfusion torniquet, the non pneumatic anti-shock garment (Life Wrap) and the non inflatable antishock garment, which are discussed in this paper.

## HISTORICAL DEVELOPMENT

Since its first use in frontline emergency care, the Military Anti-Shock Trousers (MAST) has become one of the most widely studied and debated medical devices in pre hospital care. It has enjoyed both widespread support as well as harsh criticisms. Few medical devices have engendered such divergent opinion.

The concept of the MAST was first described in 1903 by surgeon George W Crile as a pneumatic suit to decrease postural hypotension in neurosurgical patients. The resuscitation of the apparently dead and a demonstration of the pneumatic rubberised suit as a means of controlling blood pressure was reported at the meeting of The Southern Surgical and Gynaecologic Association in 1903. During World War II, Crile's suit was used to prevent blackout in pilots who were subjected to high G force while flying combat aircraft.[1–3]

The North America Space Association (NASA), then, claimed responsibility for the development of the medical anti-shock trousers at their AMES Research Centre in 1960s.[4]

MAST was subsequently introduced into medical practice during the Vietnam War and was then called the Military Anti-Shock Trousers (MAST).[5] Its value in the military was documented when soldiers with massive trauma and bleeding, previously considered fatal, were able to survive a 30-60 minute air-lift and helicopter ride to a definitive care facility.[6]

In the 1970s, MAST began to be introduced into the civilian Emergency Medical Services (EMS) systems.[7] In 1977, it was listed by the Committee on Trauma of the American College of Surgeons to be an essential device to be carried on all ambulances.[8]

It was only in 1997, based on all the available data, that the National Association of EMS Physicians (NAEMSP) issued a position paper on the use of MAST in modern EMS.[9] The association concluded that MAST was definitely beneficial in ruptured abdominal aortic aneurysm (AAA) and possibly beneficial in hypotension due to pelvic fracture, anaphylactic shock refractory to standard therapy, uncontrollable lower extremity haemorrhage and severe traumatic hypotension (palpable pulse with no blood pressure). Many EMS systems kept MAST for use in lower extremity and pelvic fracture.[10]

MAST seemed to have worked and even saved some lives then. However, today, many question whether it really worked. It is now seen as a device that takes up valuable storage space on ambulances, is expensive (at approximately USD 500 per pair) and with an uncertain future. Is the MAST a relic of our past and belong only in EMS museums rather than on EMS and rescue vehicles?

## MATERIALS AND METHODS

Literature search was conducted online with journals in OVID, MD Consult, Science Direct, Health Sciences Collection, EMBase, Medline, Cinahl, EMBR and other online nursing journals, using the following search terms: MAST trousers, MAST, Pneumatic anti-shock (PASG) garment, Anti shock trousers and pneumatic inflatable garments. The articles were reviewed and the relevant information extracted accordingly.

## MECHANISM

**Figure 1:**
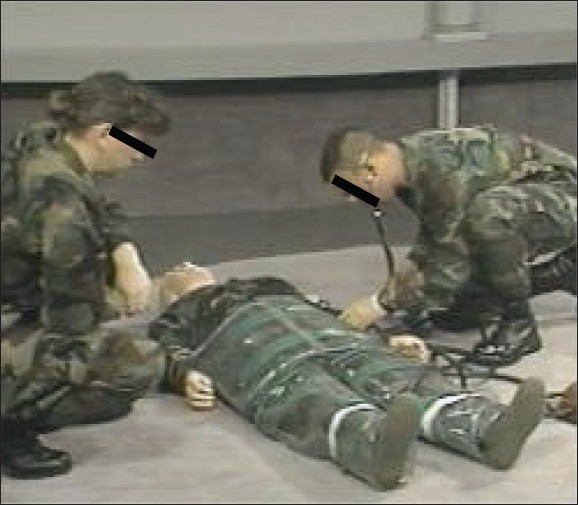
Paramedics inflating the MAST with a simulated soldier

**Figure 2:**
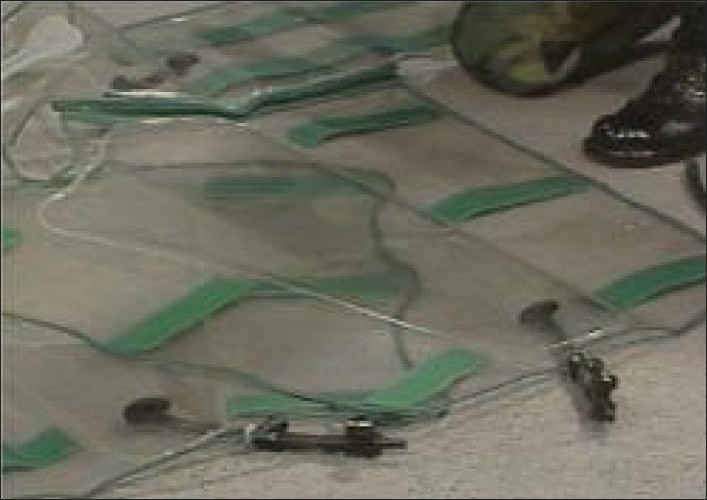
The MAST Trousers

MAST is a self -contained unit, which is made up of one abdominal compartment and two leg compartments attached to an inflation unit. It is held together by Velcro [Figures 1 and 2]. The improved MAST is constructed from urethane, which allows longer storage in the ambulances even when not in frequent use.[11]

The decision to apply the MAST should be based on the type of shock as well as the type of injury. To use the MAST, the leg compartments must be inflated first before the abdominal compartment. The stopcocks must be closed before inflation, with only one opened at a time to fill the particular compartment. The compartments should be filled until either a systolic blood pressure of 100 mmHg is reached or until the Velcro or urethane begins to pull apart. The application can be achieved by one of the three suggested methods:

**Trouser method**: The application is as though it is a pair of pants. One rescuer places his arms up through the MAST ‘legs’ to grab the patient's ankles while the other rescuers help slide the MAST up over the patient. This method should never be used when there is any possibility of spinal injury.

**Log-roll method**: The MAST is placed on a backboard and the patient is log-rolled onto the board and MAST. Once the patient is centered over the alignment markings of the MAST, wrap the legs and abdomen with the appropriate compartments and then inflated according to protocol.

**Diaper method**: The rescuers will roll the inner edges and the anterior abdominal section of the MAST towards the center of the garment and then slide the pants underneath the patient, in the same way one would slip a sheet underneath a patient. This method involves little movement and may be preferred when dealing with a hip or pelvic injury.

Deflation is rarely, if ever, done in the pre hospital setting and should always be done with direct instructions from a physician.[12]

It is postulated that the MAST reverse hypotension by at least three different mechanisms:

1. By **increasing peripheral vascular resistance,**
2. By a **tamponade effect** on intra-abdominal bleeding and
3. By **auto transfusion** of blood from the lower extremities and abdomen to the head and upper trunk.

With the mechanism of **auto transfusion**, it reduces the potential spaces where haematomas may form and supports the central circulation by redistributing blood to the vital organ systems. Auto transfusion occurs from the splanchnic bed as well. Thus, MAST application **increases the peripheral resistance** and **venous return**	 through compression of the inferior vena cava and limb vasculature.[11] The typical effect of MAST application on a normovolemic subject would include acute 100% increase of both right and left ventricular load, increase in pulmonary artery and aortic pressures by 77% and 25% respectively and an increase of the systemic vascular resistance by 22%.[13]

Studies have supported the theory that MAST application provided a significant auto transfusion. The amount of blood auto transfused is estimated to be 750 to 1000 mls.[14] Other studies estimated approximately 20% of patient's blood volume was auto transfused to the heart, brain and lungs.[15,16] It has been estimated that about 2 units of blood is being auto transfused on average. Similarly, when the MAST is released or deflated (which is not to be done in the pre hospital setting), the blood will return to the lower limbs as if the person has suddenly received two units of blood.[17] Researchers at Valley Medical Centre in Fresno, California, evaluated the effects of MAST on healthy volunteers. After the removal of one litre of blood, the MAST was applied. The amount of blood auto transfused from the lower extremities and abdomen to the head and upper trunk was measured using sequential radioisotope scans. The results showed less than 5% of the total blood volume was auto transfused. This represents only about 300 mls in an average 80 kg adult man, which is much less than the initially thought volume of 750-1000 mls.[18] This volume was also confirmed on dogs which had been phlebotomised.[18,19]

Following these studies, statements on the auto transfusion capabilities of MAST were dropped and the role of MAST was stated as only as a device that can increase the peripheral vascular resistance. The increase in the latter is a result of physiological response to loss of volume as well as a reduction in size of the enclosed vessels by external pressure. This external counter-pressure also reduces the bleeding from severed vessels beneath it. This beneficial effect is most apparent when the bleeding is due to blunt or penetrating trauma to the abdomen which results in bleeding in the abdominal and retroperitoneal regions. MAST has also been used to support and stabilize compound femoral and pelvic fractures. It helps to reduce blood loss and provide temporary splinting.[20–21]

## MAST AND THE EMS

Earlier studies done in 1995 and 1996 by an EMS survey reported that 30 states in the USA required ambulances to carry MAST. To date it is uncertain whether this figure has changed. In 1997, the National Association of EMS Physicians published a position paper in the New York State Department Advisory, pertaining to the use of MAST, based on the evaluation of 375 clinical studies and reports available then, Even though MAST use was based on medical control philosophy and not legislation, the impact of the position paper is not clearly known.[22–23]

The recommendations from the position paper were made according to various classes of evidence available [Table 1]:[23]

**Table 1:** Summary of Recommendations

**Class I**	Hypotension due to ruptured AAA
**Class II a**	Hypotesion due to suspected pelvic fracture
	Anaphylactic shock (unresponsive to standard therapy)
	Uncontrollable lower limb haemorrhage
	Severe traumatic hypotension (palpable pulse and no BP)
**Class II b**	Elderly
	History of congestive heart failure
	Penetrating abdominal injury
	Paroxysmal supraventricular tachycardia
	Gynecologic haemorrhage (otherwise uncontrollable)
	Hypothermia-induced hypotension
	Lower extremity haemorrhage
	Pelvic fracture without hypotension
	Ruptured ectopic pregnancy
	Septic shock
	Spinal shock
	Urologic haemorrhage (otherwise uncontrollable)
	Assist intravenous cannulation
**Class III**	Diaphragmatic rupture
	Penetrating thoracic CPR
	Adjunct to CPR
	Pulmonary oedema
	Extremity trauma
	Splinting fractures of lower limbs
	Abdominal evisceration
	Acute myocardial infraction
	Cardiac tamponade
	Cardiogenic shock
	Gravid Uterus

AAA: Abdominal Aortic Anuerysm, BP: Blood Pressure, CPR: Cardiopulmonary Resuscitation

Class I: most useful and effective

Class IIa: acceptable, evidence favors usefulness

Class IIb: may be helpful, probably not harmful

Class III: contraindicated and harmful

Based on this position paper, the only Class I evidence is for patients with ruptured abdominal aortic aneurysm (AAA).[23] Patients with ruptured AAA had higher survival rates with the use of MAST, together with aggressive fluid resuscitation and blood pressure control prior to surgery.[24]

Use of MAST on patients with hypotension due to suspected pelvic fracture, anaphylactic shock (unresponsive to standard therapy), uncontrolled lower extremity and severe traumatic hypotension were supported by studies as Class IIa evidence.[14,25–29] It also stated that MAST was helpful and probably not harmful (Class II b evidence) in elderly with a history of congestive hart failure, who have penetrating abdominal injuries, uncontrolled gynecologic and lower limb haemorrhage, septic and spinal shock.[23,29–31] MAST application is contraindicated (Class III evidence) in patients with diaphragmatic injury, thoracic injury, abdominal evisceration and gravid uterus among others. MAST is not used as an adjunct to Cardiopulmonary resuscitation (CPR).[23]

Prior to this position paper, the indications for use were described by McSwain:[14]

- Shock with systolic blood pressure below 80 mmHG
- Haemorrhage requiring direct pressure for control
- Leg fractures requiring immobilisation and
- Intra-abdominal hemorrhage

### Evidence: For and against

MAST raised the peripheral resistance, “analogous to the vaso-constrictive effects of adrenaline”. MAST has been shown to reverse the hypotension caused by Hymenoptera venom. These two victims of bee sting anaphylaxis survived.[27] In 1980, eleven patients with compound pelvic fractures and seven patients with various sub-diaphragmatic bleeding sites, yielded favorable survival outcomes in Netherlands.[25] Even though seven of the patients did not survive the injuries, in eleven of the cases, bleeding was arrested with the use of MAST and minimal complications were reported.[25] In 1977, forty seven patients who survived with similar injuries, were brought into the emergency department with good blood pressure with the use of MAST at scene.[13] The significance of these clinical experiences was that patients survived the subsequent surgical repair with no complication of acute respiratory distress syndrome or acute renal failure.[14]

It might also be helpful, probably not harmful (Class IIb evidence) to use MAST on patients who are elderly, with history of congestive heart failure, penetrating abdominal injuries, paroxysmal supraventricular tachycardia, uncontrolled gynecologic hemorrhage, hypothermic induced hypotension, uncontrolled lower extremity hemorrhage, pelvic fracture with or without hypotension, ruptured ectopic pregnancy, septic shock, spinal shock, uncontrolled urological hemorrhage, and assist in intravenous cannulation due to peripheral shutdown in severe hypotension cases.[23] Schou *et al.*reported improved survival rate following the use of MAST in severely hypotensive patients with abdominal trauma where survival will not be possible without the use of MAST.[28] In 1988, a case study of a lower limb crush injury of sixteen year old road traffic accident victim showed that the patient's hemodynamic parameters improved with the use of MAST, after both colloids and group O negative packed failed to improve the parameters, resulting from severe hemorrhagic shock due to a popliteal arterial injury.[30] In the arena of obstetrics and gynecology, MAST made its way into the Journal of Obstetrics, where one woman's life was saved by its use in controlling her torrential hemorrhage in an elective Cesarean delivery.[31] Subsequent studies done at Memorial Christian Hospital, Sialkot, on seven women who developed severe obstetric hemorrhagic shock (blood loss more than 250 ml/hr and mean arterial pressure less than 70 mmHg) showed six of them had their blood pressure restored and improvement of their mental status within 5 minutes from the initial presentations where two patients who were pulse-less, three were unconscious or confused, while the remaining one did not improve due to pre-existing cardiovascular condition.[32]

MAST application is contraindicated (Class III evidence) in the following patient scenarios: patients with diaphragmatic rupture, penetrating thoracic injury, abdominal evisceration and gravid uterus. It should not be used as an adjunct to CPR nor used to splint fractures of lower extremities.[23]

Patients presenting with pulmonary edema, acute myocardial infarction, cardiac tamponade, cardiogenic shock should not have MAST applied because the usage may worsen the cardiac conditions of these patients.[33]

However in the case of cardiac arrests, the contraindication to the use of it as an adjunct to CPR is debatable. In a 1983 study, it was found that in 136 cardiac arrest patients who were older than 20 years old, the survival to hospital was 9% as compared to 4% in the usual care group.[29]

In one of the obstetric hemorrhage case applications in Sialkot, one of them got the MAST removed because she suffered dyspnea, seconding to the underlying condition of mistral stenosis that was not known to the researchers and the patient herself.[32] However in trauma situations, it is difficult to isolate/exclude such patients especially if the patient is unable to give a detailed history at presentation.

Some of the contraindications mentioned above are debatable. Those that were against the use of MAST reported no or minimal usefulness to the patients, while those that suggested the use of MAST suggested the need for further studies to assess the efficacy on specific patient conditions.

Apart from contraindications, there are also disadvantages and complications which arise from the use of MAST.

MAST application prolongs scene time. EMS has been advocated to scoop and run unstable cases to reduce scene and transport time during the alleged “golden hour". Prolonged scene time for MAST application is agruable.[28] Schou J *et al.* disputed on the point of prolonged scene time. They found that 4.7 minutes deployment time at the scene was found to be realistic.[28] However, prospective studies done in 1985 had shown that use of MAST at scene do not improve the trauma score in an urban pre-hospital setting.[34] It also been said that in the preventable death evaluation study that 2 deaths were due to pre-hospital delays resulting from the use of MAST at scene.[35]

In a state or country that has air transport as part of the EMS response, emergency medicine personnel need to beware that MAST pressure is a function of altitude. In three stimulated patient transport trials in a helicopter, which ascends from 2500 to 9500 feet, MAST pressures increased respectively.[36] When the helicopter descended from 9500 feet back to 2500 feet, positive second order pressures of MAST decreased respectively.[36] The significance of this study was that MAST induced compartment syndrome may occur during transport and thus require closer monitoring.[36]

If the MAST application is prolonged, patients may suffer respiratory acidosis and decrease pulmonary vital capacity.[37] On healthy volunteers, prolonged use of MAST reduced the volunteers' forced expiratory capacity by twelve per cent, vital capacity by thirteen per cent, functional residual capacity by eighteen per cent and tidal volume by twenty five percent reductions.[38] In the case of trauma patients, one study found that out of 25 trauma patients recruited in the respiratory function study which followed application of MAST, three patients suffered severe acidosis while the rest suffered mild acidosis.[39] In the animal controlled study on pigs, hyperkalemia and lactic acidosis were reported after the prolonged use of MAST application.[40]

Another disadvantage of the use of MAST is its removal in the hospital setting. Sudden deflation is physiologically equivalent to losing a significant volume of blood in a few seconds.[41] The drastic change is in the after load distribution and the sudden flooding of the lactic acid rich blood from the lower extremities to the patient's central circulation.[41] Removal of MAST requires an experienced physician who is trained to prevent sudden loss of blood pressure to the patient.[41] However, this may cause delays as trauma surgeons and emergency physicians are unable to conduct secondary surveys and physical assessments on the MAST affected body parts whilst the MAST are inflated.[41]

These disadvantages may have contributed to the increased mortality and longer ICU stay related to the use of MAST.

MAST related compartment syndrome, leading to the total occlusion of the arterial supply of the fractured lower extremities had also been reported.[42] A review done on 27 cases in 1989 showed that MAST contributed to the process of compartment syndrome by prolonging the muscle ischemia on top of the co-morbidities of the patients' i.e. lower extremity trauma and systemic hypotension.[43] Two other studies supported the findings of MAST related compartment syndrome.[44,45] MAST was reported to cause occlusion of the iliofemoral systems from the aortic bifurcation to the feet. Ischemia of both legs and scrotum was observed in the patient who had severe hypotension secondary to nifedipine overdose. In one of the trauma angiography case studies, there was apparent total occlusion of the femoral artery revealed on thoracic angiography done on a 60 year old patient who suffered open, comminuted fractures of the right tibia and fibula and had MAST applied at scene to control blood pressure.[43] The patient had a below knee amputation during a right groin explorative operation.[43] There were two similar cases reported in emergency medicine journals that patients with lower extremity fracture suffered limb loss after use of MAST.[46,47]

MAST application trials conducted on patients with penetrating anterior abdominal injuries secondary to stab and gunshot wounds, penetrating cardiac wounds,[48] traumatic shock, haemodialysis- induced hypotension;[49] hypotension secondary to hypothermia[50] did not yield favourable outcomes. In fact, in 1987, a study found that with use of MAST on patients with anterior abdominal wounds secondary to gunshot and stab wounds, there was an 8% absolute increase in mortality at hospital discharge.[51]

However there was some success for alternative use of MAST. The haemostatic efficacy of MAST was proven in one isolated case study in 2001 on a patient with severe intra abdominal bleed from the transmural wound of the inferior vena cava.[52]

In the medical arena, there is new evidence for the use of MAST. One group is patients with pheochromocytoma or Chronic Fatigue Syndrome (CFS). These patients will have excessive orthostatic hypotension and tachycardia.[53] These patients will have excessive pooling of blood in the lower limbs resulting in subnormal compliance in the pedal veins during norepinephrine infusion. With MAST application to 30 mm Hg, there was correction of excessive lower body venous pooling and cerebral perfusion, leading to rapid improvement in symptoms. CFS affects four times more women than men. Centre of Disease Control reports an increase in the prevalence rate of CFS.[54]

In the surgical arena, the MAST were reported to have helped in stabilizing patients who had upper gastrointestinal bleed induced hypotension.[55]

The New York position paper do not recommend use of MAST on patients with induced cardiogenic shock and cardiac tamponade but animal studies apparently responded favourably to MAST application.[56] This may shed new room for further expandable research on animals and human subjects on the efficacy of MAST.

Another isolated alternative that was also looked into is the use of hypertonic saline infusion with MAST applications.[57] Results showed that it increases mean arterial pressure, thus increasing cardiac output. However, the efficacy is questionable, as isotonic solutions are advocated and more favourably recommended than hypertonic solutions.[57]

## ALTERNATIVE DEVICES

A few of alternative devices have been derived from the MAST trousers technology and these include, the auto-transfusion tourniquet (ATT), the non-pneumatic anti-Shock Garment (Life Wrap) and non-inflatable anti shock garment (NI-ASG).

ATT technology was derived from Israel Institute of Technology and marketed in the United States by OHK Medical Devices. According to the OHK product literature, the clinical indication for the use of ATT is to use in emergency situations where patients are hypotensive from hemorrhagic shock. OHK also suggests the use of ATT for hypovolemia, septic and anaphylactic shocks. The ATT is an elastic ring wrapped around by a longitudinal sleeve. When ATT is rolled around a limb, it compresses the tissues beneath it with a pressure that is higher than the systolic blood pressure and remains as an arterial tourniquet at the proximal end of the limb.[58–60] The physiological and biochemical effects of ATT on 18 normal volunteers were assessed by applying the ATT device on them for 20 minutes. It was observed that ATT gave rise to 17.4; (14.5-20.3) (Mean; (CI)) and 13.1; (11.0-15.1) mm Hg rise in systolic and diastolic blood pressures, respectively (*P* < 0.0001). The leg volume dropped by 1040 ml; (863-1216) ml during ATT application (*P* < 0.0001). Ventilation and gas exchange parameters did not change except for a slight rise in S_p_ O_2_(*P* < 0.001). There was a statistically significant decline in creatinine phosphokinase (CPK) and lactic acid, with no change in Ca^++^ and Na^+^ concentrations. The authors concluded that ATT indeed induces significant auto-transfusion and blood pressure elevation in normal volunteers.[58] Moreover the biochemical data indicated that applying the ATT did not cause significant deleterious tissue ischemia or mechanical crush injury.[53] The authors have recommended that the usefulness of ATT to be further evaluated in the actual treatment of shock victims.

With maternal mortality rate of over 500,000 deaths globally each year, obstetric haemorrhage contributed to a large proportion of the deaths. Obstetric hemorrhage requires definitive care and early surgery with blood transfusion.[59] However, in rural areas, provinces or states, definitive care can be delayed. Life Wrap offered a possible solution of first aid to arrest bleeding. The Life Wrap is a simple neoprene and Velcro device much like the bottom half of a wet suit split down the middle and it is used to treat massive post partum haemorrhage. Life Wrap could be effectively deployed by any trained staff to render “obstetrical first aid" to a post partum hemorrhaging woman prior to transfer. Life Wrap provides continuous and safe lower body counter pressure to effect resuscitation and arrest further bleeding. The counter pressure not only returns blood to the vital organs but also slows the blood flow to the lower body and decrease bleeding tendency. This works along the same lines of the MAST principle of “auto transfusion”.[59]

Life Wrap studies have been conducted in Mexico and Nigeria, with the goal of determining the effectiveness and safety of the device to prevent maternal mortality in both rural and urban areas. Preliminary results showed that there is a 50% reduction in bleeding and 75% women were less likely to die from obstetric haemorrhage.[59]

Another obstetric hemorrhage rescue device that is on trial in Mexico, Egypt and Nigeria is the NI-ASG device. NI-ASG is a low technology, light weight, reusable compression suit made from Neoprene. It has horizontal panels at the lower limbs and the abdominal panel is secured closely by Velcro.[61] The garment is applied to obstetric patients with hemorrhagic shock. The NI-ASG produces a circumfential counter pressure evenly distributed throughout the abdominal cavity and to the outside of the circulatory vessels of the patients. Such pressure leads to reduction of the transmural pressures and lowers the tension in the arterial wall, and closure of the opening of the blood vessels, resulting in haemostasis. The benefits of the NI-ASG garment were numerous. Firstly, the garment can be applied by any person regardless of educational background and medical training. It could be applied by a solo care provider or lay person. Secondly; the NI-ASG garment can remain safely on the obstetric patients for more than two days with no reported complications. Thirdly, the garment significantly reduces the blood loss without the need for interventional procedures such as uterine packing, ligation of vessels or hysterectomy. Lastly, the pressure of only 20-40 mm Hg would be a low risk for causing ischemia or necrosis in the lower body.[61]

## CONCLUSION

The MAST continues to be used only very rarely today. The younger generation of healthcare providers may have never seen it, but it will continue to be in our medical archives surely, for the impact it may have had and the discussions generated during its hey days. Research done over the years have showed both positive as well as negative results to support its use as a standard of care. MAST may never relive the glory it has had, but we have had the priviledge of having that option to be able to choose it when deemed necessary. As with other devices, evolution and fine tuning occurs; devices with higher level of sophistication, high usage of technology, very rapid deployment continues to be invented and developed.
